# p63 regulates cell proliferation and cell cycle progression-associated genes in stromal cells of giant cell tumor of the bone

**DOI:** 10.3892/ijo.2012.1727

**Published:** 2012-12-03

**Authors:** CAROL PO YING LAU, PATRICK KWOK SHING NG, MAN SHAN LI, STEPHEN KWOK WING TSUI, LIN HUANG, SHEKHAR MADHUKAR KUMTA

**Affiliations:** 1 Department of Orthopaedics and Traumatology, The Chinese University of Hong Kong, Shatin, N.T., Hong Kong, SAR, P.R. China; 2 School of Biomedical Sciences, The Chinese University of Hong Kong, Shatin, N.T., Hong Kong, SAR, P.R. China; 3 Division of Plastic, The Chinese University of Hong Kong, Shatin, N.T., Hong Kong, SAR, P.R. China

**Keywords:** giant cell tumor of bone, p63, CDC2, CDC25C, cell proliferation, siRNA

## Abstract

Giant cell tumor of bone (GCT) is a destructive neoplasm of uncertain etiology that affects the epiphyseal ends of long bones in young adults. GCT stromal cells (GCTSCs) are the primary neoplastic cells of this tumor and are the only proliferating cell component in long-term culture, which recruits osteoclast-like giant cells that eventually mediate bone destruction. The oncogenesis of GCT and factors driving the neoplastic stromal cells to proliferate extensively and pause at an early differentiation stage of pre-osteoblast lineage remain unknown. Overexpression of p63 was observed in GCTSCs and there is growing evidence that p63 is involved in oncogenesis through different mechanisms. This study aimed to understand the specific role of p63 in cell proliferation and oncogenesis of GCTSCs. We confirmed p63 expression in the mononuclear cells in GCT by immunohistochemical staining. By real-time PCR analysis, we showed a higher level (>15-fold) of TAp63 expression in GCTSCs compared to that in mesenchymal stem cells. Furthermore, we observed that knockdown of the p63 gene by siRNA transfection greatly reduced cell proliferation and induced cell cycle arrest at S phase in GCTSCs. We found that the mRNA expression of CDC2 and CDC25C was substantially suppressed by p63 knockdown at 24–72 h. Moreover, p63 was found to be recruited on the regulatory regions of CDC2 and CDC25C, which contain p53-responsive elements. In summary, our data suggest that p63 regulates GCTSC proliferation by binding to the CDC2 and CDC25C p53-REs, which may inhibit the p53 tumor suppressor activity and contribute to GCT tumorigenesis.

## Introduction

Giant cell tumor of bone (GCT) is a destructive neoplasm of uncertain etiology that affects the epiphyseal ends of long bones in young adults. The tumor causes severe bone destruction in the vicinity of major skeletal joints necessitating complex reconstructive surgery to eradicate the tumor and save the joint. Despite aggressive therapy, this tumor tends to recur locally, eventually requiring surgical measures of increasing complexity, resulting in significant morbidity and disadvantages to these young patients. Histologically this tumor epitomizes the model of bone destruction brought about by pathological processes. GCT exhibits three histological different cell types (1): the multinucleated osteoclast-like giant cells, the spindle-shaped stromal-like cells and the round-shaped macrophage-like cells. GCT stromal cells (GCTSCs) are the primary neoplastic cells of this tumor and are the only proliferating cell component in long-term culture (2), which recruits osteoclast-like giant cells that eventually mediate the bone destruction. GCTSCs are able to proliferate unlimitedly in cell culture and express early osteoblastic differentiation markers such as collagen type I, bone sialoprotein and osteonectin proteins (3). The oncogenesis of GCT and what drives the neoplastic stromal cells to proliferate extensively and pause at an early differentiation stage of preosteoblast lineage remains unknown.

Telomeric associations (TAS) have been reported as one of the most common genetic aberrations in GCT (4–6). It has been proposed that telomeric instability is responsible for a large degree of intra-tumor heterogeneity and the telomere serves as a precursor lesion to subsequent clonal structural aberrations of chromosome 11 in GCT. Altered telomerase (hTERT) activity and inactivation of an alternative telomere lengthening mechanism were found in GCT, suggesting that TAS alone does not contribute to the development of genetic instability in GCT (7). The cause of genetic instability in GCT remains obscure. In a recent study it was shown that p63 expression is significantly higher in GCT than many other primary bone tumors (8). Another report demonstrated the expression of TAp63 isoform but not DNp63 in GCT mononuclear cells (9).

p63 gene encodes two primary transcripts, TAp63 and DNp63, which are controlled by two separate promoters, P1 and P2. TAp63 is generated from the P1 promoter and contains the transactivation domain (TAD), the DNA binding domain (DBD) and the oligomerization domain (OD). In contrast, DNp63 is generated from the P2 promoter and does not have an amino terminal TAD. The DN-terminal variants are generally regarded as dominant negative versions of p53 family members, as they can occupy promoter-binding sites but fail to transactivate gene expression (10,11). Independent of their N-terminal status, p63 exists as a variety of C-terminal splicing variants, most commonly known as three variants, α, β and γ (12). In addition, two more C-terminus p63 variants have been discovered, the variant δ derived from the skipping of exons 12 and 13, and the variant ε generated by a premature transcriptional termination in intron 10 (13). p63 is required for limb and skin development (14,15). It is also involved in modulation of gene expression associated with apoptosis, cell proliferation and inhibition of tumor progression in p53-dependent signaling pathways. Moreover, p63 can also regulate gene expression in p53-independent pathways for more specific genes that are associated with development, epithelial terminal differentiation and cell adhesion. Since there is growing evidence that p63 is involved in oncogenesis through different mechanisms, this study aimed to understand the specific role of p63 in cell proliferation and oncogenesis of GCTSCs.

## Materials and methods

### Cell culture

GCT specimens were collected from the patients underwent surgical excision of the tumor at Prince of Wales Hospital. All protocols were approved by the local institutional ethics committee. Primary culture of GCTSCs was established as described previously (16). In brief, freshly obtained GCT tissues were chopped in DMEM containing 10% FBS and 100 U/ml penicillin (PSN). The resultant cell suspension was transferred to culture flasks and cultured at 37°C in a humidified atmosphere of 5% CO_2_ and 95% air. Culture medium was changed every 2–3 days and upon reaching confluence, the cells were subcultured. The stromal phenotype of GCTSCs was verified by immunofluorescent staining using mouse anti-human STRO-1 monoclonal antibody (MAB4315; Chemicon International, Temecula, CA, USA). Mesenchymal stem cells (MSC) obtained from bone marrow of healthy donors were used as the control cells for evaluation in this study. GCTSC has been reported to be of the MSC origin (17,18). Bone marrow aspirated from the iliac crests was collected and the mononuclear cells were isolated by Ficoll gradient centrifugation method. The cells were rinsed twice with PBS and cultured in DMEM containing 2% FBS and 100 U/ml penicillin (PSN). The cells were then cultured at 37°C in a humidified atmosphere of 5% CO_2_ and 95% air.

### Immunohistochemistry

GCT specimens collected from the patients were fixed, embedded and processed for tissue sectioning. For immunohistochemical staining of p63, tissue sections (5 *μ*m) were de-waxed in xylene and then rehydrated in sequentially diluted ethanol followed by PBS. Endogenous peroxidase activity was then blocked with 0.3% hydrogen peroxide in absolute methanol for 20 min. Tissue sections were then incubated with 5% normal goat-serum in 1% BSA-PBS for 30 min to block non-specific IgG binding. Thereafter, primary antibody p63 (Abcam, Cambridge, UK) was applied at a dilution of 1:500 in 1% BSA-PBS for incubation at 4°C overnight. After stringent washing twice in PBS, a secondary antibody was used for further incubation and a streptavidin-biotin complex system (ABC reagent, Dako, Ely, Cambridgeshire, UK) with diaminobenzidene (DAB) as chromogen was used for color development. Slides were finally counterstained with hematoxylin and examined under a light microscope.

### p63 knockdown by siRNA transfection

GCTSCs were plated into 6-well plates at a density of 1.5×10^5^ cells/well or 96-well plates at 7,000 cells/well depending on the types of assessment to be performed following siRNA transfection. The cells were kept in antibiotic-free medium for 24 h before transfection. The cells were then transfected with a mixture of OptiMEM, 5 *μ*l of lipofectamine/well (Lipofectamine™ RNAiMAX Transfection Reagent, Invitrogen, Grand Island, NY, USA) and either scramble siRNA (Stealth RNAi™ siRNA Negative Controls, Invitrogen) or p63 siRNA (TP63 Stealth Select RNAi™ siRNA, Invitrogen) at a final concentration of 50 nM for 48 to 72 h. The sequences of these siRNAs are available from the manufacturer. The cells were then subjected to further assessments as follows.

### Bromodeoxyuridine (BrdU) incorporation assay and cell cycle analysis

Cell proliferation rate at 72 h after transfection was measured by BrdU incorporation using the Cell Proliferation ELISA, BrdU (colorimetric) kit (Roche Diagnostics, Germany) according to the manufacturer’s instructions. In a parallel experiment for cell cycle analysis, the transfected cells were trypsinized, washed twice with PBS and fixed in 70% ethanol. The fixed cells were washed with PBS, incubated with 100 mg/ml RNase at 37°C for 30 min, stained with PI (50 mg/ml) and analyzed on a FACScan flow cytometer (Becton-Dickinson, USA). The percentages of cells in different phases of the cell cycle were analyzed using the ModFit LT version 3.0 (Verity Software House, Topsham, ME, USA).

### mRNA extraction and real-time PCR

The cells were harvested at 48 h after transfection for RNA extraction with TRIzol^®^ reagent (Gibco-BRL, Grand Island, NY, USA). Extracted RNA was reverse transcribed into first-strand cDNA using QuantiTect Reverse Transcription kit (Qiagen, Valencia, CA, USA). The amount of cDNA used for the amplification of the target genes were normalized by human GAPDH gene. Primer sequences have been designed using Primer Express from Applied Biosystems (Branchburg, NJ, USA). The ABI 7500 fast real-time PCR system and power SYBR-Green PCR Master mix (Applied Biosystems) were used to perform 40 cycles of PCR with each PCR performed in triplicate.

### Protein extraction and western blot analysis

Total cell lysates from the transfected cells were denatured by boiling in Laemmli sample buffer and loaded onto a gradient SDS-polyacrylamide gel (30 *μ*g protein/lane). After being resolved by electrophoresis, proteins were transferred to an Immobilon-P membrane (Millipore, Bedford, MA, USA) via electroblotting. The membrane was blocked with 5% non-fat milk in TBST and incubated overnight at 4°C with specific primary antibodies. p63, CDC25C and CDC2 antibodies (Abcam, Cambridge, UK) were used, followed by incubation with an anti-mouse IgG secondary antibody conjugated with horseradish peroxidase (Pierce, Rockford, IL, USA). Sites of antibody-antigen reaction were visualized by using the SuperSignal West Pico Chemiluminescent Substrate kit (Pierce) and recorded by a Kodak 440CF Image Station.

### Chromatin immunoprecipitation (ChIP) assay

Chromatin immunoprecipitation assay was performed using ChampionChIP One-Day Kit (SABiosciences, Valencia, CA, USA) according to manufacturer’s instruction with few modifications as described below. Briefly, GCTSCs were seeded in 100-mm dish and harvested until 80–90% confluence. Chromatins were prepared and fragmented by sonication. Fragmented chromatin was pre-cleared with protein A beads and normal mouse IgG (Santa Cruz Biotechnology, Santa Cruz, CA, USA) at 4°C for 2 h. Ten microliters pre-cleared lysate was saved up as input fraction. Two immunoprecipitation reactions were set up with 1 ml pre-cleared lysate and 4 mg normal mouse IgG or anti-p63 antibody (Abcam). Reactions were incubated at 4°C overnight and followed by adding 60 *μ*l of protein A beads and incubated at 4°C for further 1 h. After a number of washing steps, reverse cross-linking and purification of pulldown chromatins, as well as input fraction, were performed as described in manufacturer’s manual. The immunoprecipitated material was amplified using primers specific for CDC2 promoter: 5′-AGCCTCTTTCTCTC CCCTCATAGA-3′ (forward), 5′-AGAGGCATGCATTTAGAG ACTG-3′ (reverse) or CDC25 promoter: 5′-ATTCGGCCCTCC CAACCTCTGT-3′, 5′-GTCTTCGCCTGTGTCCGATCCC-3′ (reverse) which contain the p53-reponsive elements (p53-REs).

## Results

### p63 expression in GCT

**Figure 1.**
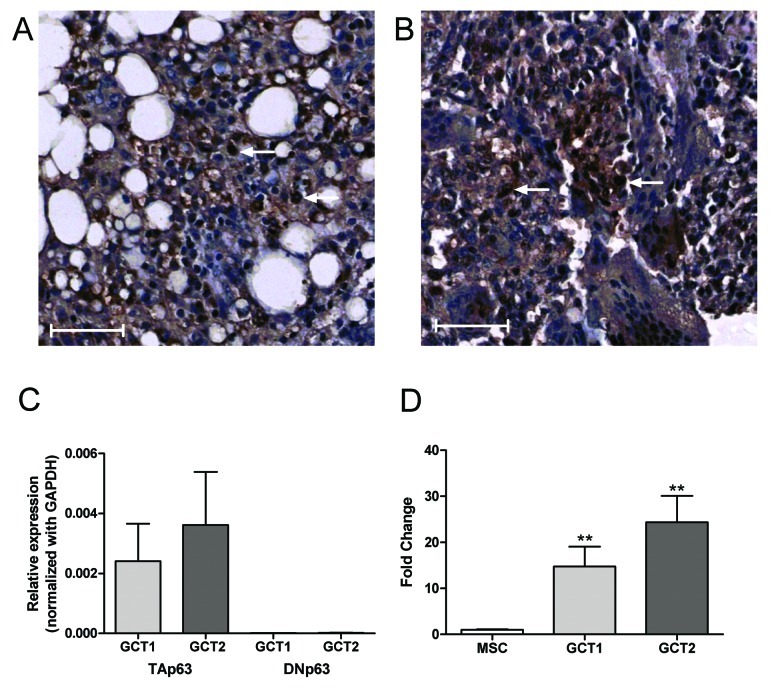
p63 protein and gene expression in GCT. (A and B) Immunostaining for p63 in paraffin-embedded tissue sections of two representative cases of GCT showed expression mainly in mononuclear cells (arrows); scale bar, 50 *μ*m. (C) TAp63 isoform was detected in the cell lines and DNp63 was at undetectable level. (D) GCTSCs overexpressed TAp63 (>15-fold) when compared with MSCs; error bars represent SD of three independent experiments, ^**^p<0.001 in Dunnett’s multiple comparison test.

**Figure 2.**
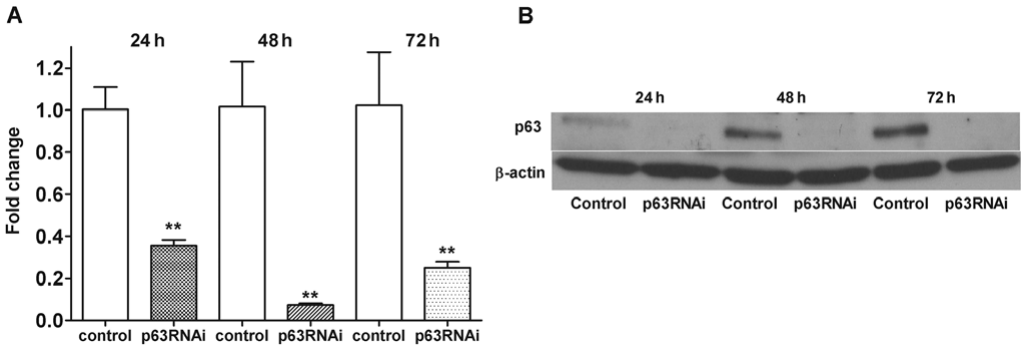
p63 siRNA abrogates both (A) mRNA and (B) protein expression of p63 in GCTSC. GCTSCs were treated with either control siRNA (control) or p63 siRNA (p63RNAi) from 24–72 h. Gene expression was assessed by real-time PCR, p<0.01 by Student’s t-test and protein level was confirmed by western blot analysis. Expression of β-actin is shown for comparison.

By immunohistochemical staining, p63 expression was found to be mainly localized in the nuclei of mononuclear cells in GCT specimens (Fig. 1A and B). By quantitative real-time PCR analysis, TAp63 isoform, but not DNp63 was detected in GCTSCs from primary culture (Fig. 1C). As compared to bone marrow-derived MSCs, the expression of TAp63 mRNA in GCTSC was significantly higher (>15-fold) (Fig. 1D). In order to determine the functional role of endogenous p63 in GCTSC, we used a RNA-mediated interference (siRNA) approach to knockdown p63 in GCTSCs and assessed the morphological changes and cell proliferation status associated with p63 suppression in GCTSCs. The capability of p63 siRNA to abrogate the endogenous p63 expression in GCTSCs at 24–72 h was confirmed by real-time PCR (Fig. 2A) and western blot analysis (Fig. 2B). The level of p63 mRNA expression was reduced to 0.07-fold of the control at 48 h.

### Knockdown of p63 inhibited cell proliferation and induced cell-cycle arrest

**Figure 3.**
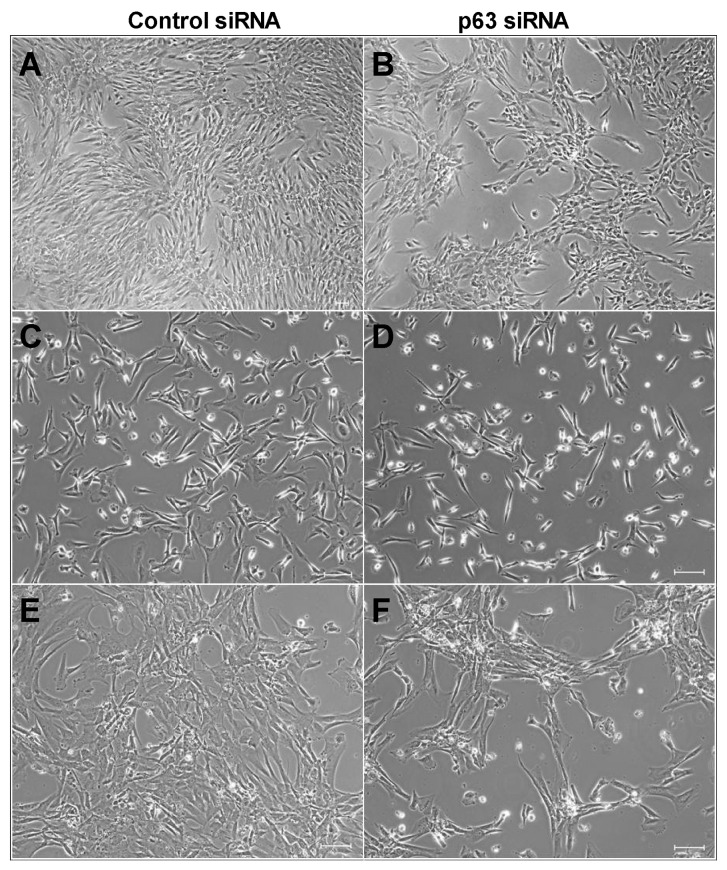
(A and B) Phrase contrast micrographs showing the morphology of MSCs and (C–F) two different GCTSC cell lines were transfected with either (A, C and E) control siRNA or (B, D and F) p63 siRNA for 72 h. Suppression of endogenous p63 reduced cell density; scale bar represents 100 *μ*m.

**Figure 4.**
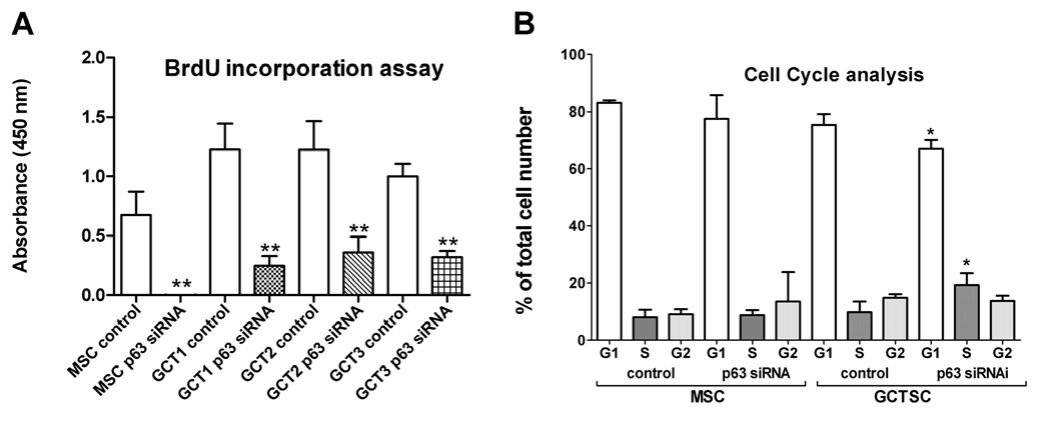
Suppression of p63 expression greatly reduced cell proliferation and induced S-phase arrest in GCTSC. MSCs and GCTSCs were transfected with control siRNA or p63 siRNA, cell proliferation was measured by (A) BrdU incorporation assay after 72 h, ^**^p<0.01 compared with control by Student’s t-test. The cell cycle analysis was performed with Modfit 3.0 (Becton-Dickinson). (B) A representative graph is shown from three independent experiments, ^*^p<0.05 compared with control by Student’s t-test.

Suppression of p63 greatly reduced cell density in both MSCs and GCTSCs at 72 h after siRNA transfection (Fig. 3). Further examination of cell proliferation and apoptosis by BrdU assay and Annexin V/PI staining followed by flow cytometry analysis, respectively, showed that p63 knockdown significantly inhibited cell proliferation in both MSCs and GCTSCs (Fig. 4A). It was noted that p63 siRNA completely ceased the cell proliferation in MSCs but inhibited 68 to 80% of cell proliferation in GCTSCs. No significant cell apoptosis was observed after p63 suppression in both MSCs and GCTSCs (data not shown). In addition, suppression of p63 led to cell cycle arrest at S-phase in GCTSCs. The percentage of cells in S-phase was significantly increased at 72 h after p63 siRNA transfection (Fig. 4B). Knockdown of p63 in MSCs increased the cell population at G2-phase, but not to a significant level.

### Suppression of p63 reduced cell cycle progression-associated gene expression

**Figure 5.**
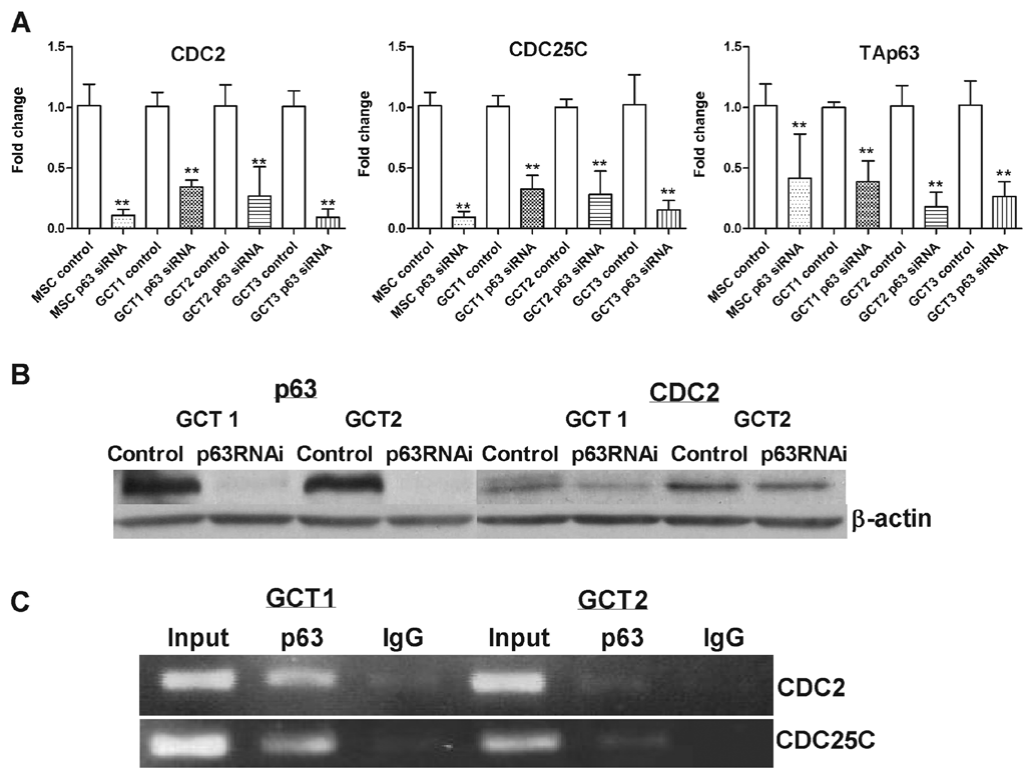
(A) Suppression of p63 expression reduced CDC2 and CDC25C gene expression. MSCs and GCTSCs were transfected with control or p63 siRNA. The mRNA expression of TAp63, CDC2 and CDC25C was evaluated by real-time PCR. Error bars represent SD of three independent experiments, ^**^p<0.01 compared with control by Student’s t-test. CDC2 protein level was also downregulated by p63 siRNA treatment at 48 h in GCTSCs and measured (B) by western blot analysis. (C) *In vivo* binding of p63 to the regulatory regions of CDC2 and CDC25C genes. Formaldehyde cross-linked chromatin was extracted from two different GCTSC cell lines and immunoprecipitated with the p63 antibody. DNA was extracted and analyzed by PCR. One representative result of three independent experiments is shown.

Further examination of cell cycle progression-associated genes including ADA, CCND3, POLD2, CDC25C and CDC2 were performed, the selection was on the basis of their reported function associated with transcriptional regulation of the cell cycle (19). The mRNA expression of CDC2 and CDC25C in p63-siRNA-transfected cells were found substantially reduced after 24–72 h treatment. This finding was verified in three GCTSC cell lines as well as MSCs (Fig. 5A). p63 siRNA-mediated downregulation of CDC2 at protein level was confirmed by western blot analysis (Fig. 5B), however, CDC25C protein was not detected in GCTSCs in the assay (data not shown).

### In vivo binding of p63 to the regulatory regions of CDC2 and CDC25C genes

A chromatin immunoprecipitation assay was performed to correlate the transcription of CDC2 and CDC25C genes with the *in vivo* recruitment of p63 on their regulatory regions. Cross-linked chromatin from two GCTSC cell lines were immunoprecipitated with a specific anti-p63 antibody (Fig. 5C). p63 was bound to the p53-RE present in the regulatory region of CDC2 and CDC25C genes.

## Discussion

In the present study, we confirmed p63 expression in the mono-nuclear cells in GCT, which is consistent with the data reported elsewhere (7,8). By quantitative real-time PCR analysis, we showed a higher level (>15-fold) of TAp63 expression in GCTSCs than that in MSCs. Furthermore, we observed a 75% knockdown of p63 gene in GCTSCs by siRNA transfection at 72 h, which led to a greatly reduced cell proliferation rate to 20 to 32% of the control in three GCTSC cell lines examined. Suppression of endogenous p63 induced cell cycle arrest at S-phase in GCTSCs. These findings suggest that p63 may regulate specific genes associated with cell cycle progression. Thus a number of cell cycle associated genes regulating the tumor suppressor activity of p53 were further investigated. It was found that the mRNA expression of CDC2 and CDC25C was substantially reduced by p63-siRNA transfection for 24–72 h. By ChIP assay, p63 was found to be recruited on the regulatory regions of both CDC2 and CDC25C genes which contain the p53-REs in GCTSCs. The *in vivo* binding of p63 to both CDC2 and CDC25C p53-REs in GCTSC suggests that p63 may promote GCT progression by inactivating the tumor suppressor activity of p53.

TAp63 was also found to inactivate the tumor suppressor activity of p53 in thyroid cancer and promotes cancer progression (20). There has been growing evidence that p63 is involved in oncogenesis through different mechanisms. For example, p63 mediates survival in squamous cell carcinoma by suppression of p73-dependent apoptosis (21); it also contributes to tumori-genesis by conferring a proliferative potential on cancer cells, allowing increased self-renewal by transactivating target genes responsible for cell division such as the adenosine deaminase gene (22). In addition, an imbalance in the expression of TA and DN isoforms for the benefit of DNp63 variants has been reported in squamous cell carcinoma of the nasopharynx (23), skin (24), lung (25), bladder (26) and oesophagus (27). The overexpression of DNp63 in many cancers (28,29) provides evidence that DNp63 may be a true oncogene in several tumor types. Nevertheless, a previous report showing TAp63 expression in gastric cancer suggests that TAp63 may also be involved in tumor progression (30). The detailed mechanisms of p63 in GCT tumorigenesis, however, remain obscure at the molecular level; further studies on the regulation of gene transcription of CDC2 and CDC25C and inactivation of p53 are necessary to further validate our hypothesis.

In summary, this study demonstrated that p63 regulated cell proliferation through two cell cycle related genes, CDC2 and CDC25C, by binding to their p53-REs in human GCTSCs. This provides a potential molecular rationale for treating GCT through targeting p63 gene. Knockdown of the overexpressed p63 by gene delivery for inhibiting the neoplastic stromal cell proliferation may provide a novel therapeutic strategy for GCT. The significant advantage of the gene therapy is that it may reduce the proliferation rate of the neoplastic GCTSCs and attenuate the aggressiveness of the GCT, resulting in less bone destruction. This may enable clinicians to scale back the extent of surgery, thereby preventing the unnecessary sacrifice of major bones and joints. Indeed, our group has recently pioneered the minimally invasive approach towards GCT treatment. This gives a major clinical benefit to GCT patients as it reduces the need for major surgery and enables the delivery of potential adjuvant drugs directly into the tumor cavity (31).
